# Emerging Roles of Ferritinophagy, Mitophagy and Chaperone-Mediated Autophagy in Pathogen-Induced Ferroptosis During Mastitis

**DOI:** 10.3390/antiox15101271

**Published:** 2026-10-02

**Authors:** Muhammad Zahoor Khan, Mengmeng Li, Abdul Qadeer, Abd Ullah, Junjie Liu, Qingshan Ma, Wenqiang Liu, Changfa Wang

**Affiliations:** 1 College of Agriculture and Biology, Liaocheng University, Liaocheng 252000, China; zahoorkhan@lcu.edu.cn (M.Z.K.);; 2 School of Medical Sciences, Shandong Xiehe University, Jinan 250109, China

**Keywords:** ferroptosis, mastitis, mammary epithelial cells, GPX4, Nrf2, lipid peroxidation, ferritinophagy, chaperone-mediated autophagy, iron homeostasis, mastitis resistance breeding

## Abstract

Mastitis, driven predominantly by *Staphylococcus aureus*, *Escherichia coli*, *Klebsiella pneumoniae* and *Streptococcus* species, remains a leading cause of economic loss and antimicrobial use in dairy production. Growing evidence implicates ferroptosis—an iron-dependent, non-apoptotic cell death executed through uncontrolled membrane lipid peroxidation—as a central mechanism of mammary epithelial injury during intramammary infection. Synthesizing evidence from bovine, caprine and murine systems, this review shows that phylogenetically diverse mastitis pathogens converge on a shared three-pillar ferroptotic program: autophagic liberation of catalytic iron via ferritinophagy, mitophagy and chaperone-mediated degradation of glutathione peroxidase 4 (GPX4); lipid-metabolic remodeling that expands peroxidation-susceptible phospholipid substrates; and collapse of the system Xc^−^/glutathione (GSH)/GPX4 antioxidant axis under Nrf2 control. Viral, xenobiotic, nanomaterial and microbiota-driven insults all engage the same program. The endpoint is convergent, not pathogen-specific. Anti-ferroptotic interventions vary in mechanism, from phytochemicals and selenium nanomaterials to extracellular vesicles and engineered stem cells. Most act on the same few regulatory nodes, chiefly Nrf2, SIRT1, SLC7A11 and GPX4. Ferroptosis-associated genes are being tested as biomarkers for resistance breeding. We grade studies by how directly ferroptosis was shown, separating direct evidence from inference based on upstream omics alone. Questions remain about cell-type-specific roles, timing during natural infection, and whether models translate to the field.

## 1. Introduction

### 1.1. Mastitis: Etiology and Clinical Significance

Mastitis, the inflammation of the mammary gland, remains one of the most prevalent and economically damaging diseases affecting the global dairy industry [1,2]. In dairy cattle, the disease compromises animal welfare, reduces milk yield and quality, increases culling rates, and imposes an enormous financial burden, with annual losses estimated in the tens of billions of dollars worldwide [3]. It is most frequently triggered by intramammary bacterial infection, with *Staphylococcus aureus*, *Escherichia coli*, *Klebsiella pneumoniae*, *Streptococcus* species, and non-aureus staphylococci among the predominant etiological agents, and it presents in both clinical and subclinical forms [4,5,6]. For decades, antibiotic therapy has served as the cornerstone of both prophylaxis and treatment; however, its effectiveness is increasingly undermined by low cure rates, the emergence of multidrug-resistant and methicillin-resistant strains, the risk of antimicrobial residues in milk, and mounting public-health concerns surrounding antimicrobial resistance [7,8,9,10]. These constraints have shifted attention toward host-directed strategies that limit tissue injury rather than targeting the pathogen alone, an approach that requires a precise account of the cellular and molecular events governing mammary tissue destruction during infection [11,12,13].

Mastitis destroys mammary epithelial cells. That loss disrupts the blood–milk barrier, amplifies inflammatory signaling, and impairs secretory function [14]. The classical cascade runs through Toll-like receptors, nuclear factor-κB, and pro-inflammatory cytokines such as TNF-α, IL-1β, and IL-6. But accumulating evidence now points to several forms of regulated cell death including Apoptosis, necroptosis, pyroptosis, autophagy, and ferroptosis which shape disease progression and severity [14]. Among these, ferroptosis has attracted particular attention, because its two defining requirements—catalytic iron and failure of GSH-dependent peroxide clearance—are precisely the conditions that bacterial infection imposes on the mammary gland [15].

### 1.2. Ferroptosis, Mitophagy and Autophagy, and Their Emerging Links to Mastitis

Ferroptosis is a non-apoptotic, iron-dependent form of cell death. It follows inhibition of GSH biosynthesis or of the GSH-dependent antioxidant enzyme glutathione peroxidase 4 (GPX4). The hallmarks are accumulation of lipid-reactive oxygen species and depletion of plasma-membrane polyunsaturated fatty acids [16,17]. Iron catalyzes the buildup of lethal lipid peroxides in cellular membranes when oxidative damage outpaces antioxidant defense [17,18]. Execution needs three conditions at once: a pool of redox-active iron; a membrane enriched in peroxidation-susceptible polyunsaturated phospholipids, made largely through acyl-CoA synthetase long-chain family member 4 (ACSL4); and failure of the antioxidant axis that detoxifies lipid hydroperoxides. That axis is the system Xc^−^/glutathione/GPX4 module. The cystine/glutamate antiporter system Xc− (SLC7A11) imports cystine for GSH synthesis, and GPX4 then uses GSH to reduce phospholipid hydroperoxides to non-toxic lipid alcohols [17,19]. This defense fails through cysteine deprivation, GSH depletion, GPX4 inactivation, or iron overload that fuels the Fenton reaction. Then, the polyunsaturated fatty acids in membrane phospholipids undergo uncontrolled peroxidation, and the membrane ruptures [18,19]. Characteristic morphological hallmarks include shrunken mitochondria with condensed membrane densities and diminished or absent cristae, while the program as a whole is placed under transcriptional control by nuclear factor erythroid 2-related factor 2 (Nrf2), with parallel inputs from the FSP1–CoQ10 axis further modulating cellular susceptibility [18].

In the bovine mammary gland, these mechanisms are increasingly implicated in the pathophysiology of mastitis. During intramammary infection, invading pathogens such as *Staphylococcus aureus*, *Escherichia coli*, and *Streptococcus* species, together with their associated virulence factors, provoke a pronounced inflammatory response in mammary epithelial cells that is accompanied by excessive reactive oxygen species production and disrupted iron homeostasis—conditions that favor lipid peroxidation and ferroptotic cell death [20]. The resulting loss of mammary epithelial integrity compromises the blood–milk barrier, exacerbates local inflammation, and contributes to the tissue damage and reduced milk yield characteristic of clinical and subclinical mastitis in dairy cattle, positioning the system Xc^−^/glutathione/GPX4 axis and its regulatory partners as promising targets for mitigating inflammation-associated injury and preserving mammary gland function in affected animals. Consistent with this premise, a growing body of evidence now directly implicates ferroptosis in mastitis pathogenesis. *S. aureus* has been shown to induce ferroptosis in mammary epithelial cells through endoplasmic reticulum stress-mediated autophagy, leading to ferritin degradation and iron dysregulation, with the ferroptosis inhibitors ferrostatin-1 and deferiprone conferring marked protection [21]. Similarly, *K. pneumoniae* induces ferroptosis in bovine mammary tissue by suppressing the Nrf2/xCT/GPX4 pathway, while lipopolysaccharide- and interleukin-6-driven models reveal ferroptotic injury that impairs milk synthesis [15,22]. Ferroptosis-related genes such as HMOX1 and SAT1 have also been proposed as molecular markers of mastitis susceptibility, and a range of bioactive natural compounds—including curcumin, schisandrin B, caffeic acid, and chlorogenic acid—appear to alleviate mammary inflammation by modulating ferroptotic signaling [14,23,24]. Collectively, these findings position ferroptosis not merely as a passive consequence of infection but as a biologically actionable therapeutic target.

### 1.3. Aim, Scope and Structure of This Review

A recent review has framed ferroptosis in bovine mastitis as a candidate mechanism of host-damage amplification and has proposed evidentiary criteria for attributing causality in complex infection models [20]. That contribution establishes an important standard of interpretive caution, and a comparable standard is adopted here. The present review is nonetheless distinct in scope and organizing logic. First, it identifies autophagic liberation of sequestered iron as the unifying upstream event across phylogenetically diverse pathogens, showing that endoplasmic reticulum stress-driven ferritinophagy in *S. aureus* infection, Wnt/β-catenin-driven mitophagy in *E. coli* infection, NCOA4-mediated ferritinophagy in *K. pneumoniae* infection, and chaperone-mediated autophagy of GPX4 in *Streptococcus uberis* infection represent four routes to a single biochemical endpoint. Second, it provides a systematic pharmacological synthesis, cataloging more than twenty phytochemical, nutritional, selenium-based and cell-based interventions together with their molecular targets, and demonstrating that these mechanistically diverse agents converge on a small set of regulatory nodes—chiefly Nrf2, SIRT1, GPX4 and SLC7A11—rather than acting through compound-specific mechanisms. Third, it evaluates ferroptosis-associated genes as candidate markers for mastitis-resistance breeding, thereby linking mechanistic insight to genetic improvement. Fourth, it examines whether the proposed mechanism holds across experimental systems rather than within a single model, drawing on murine mastitis models, immortalized and primary bovine mammary epithelial cells, and naturally infected mammary tissue, together with caprine systems for those pathways where bovine data are not yet available, most notably chaperone-mediated autophagy of GPX4. It further extends beyond classical bacterial infection to viral, xenobiotic, nanomaterial and microbiota-driven triggers, testing whether the same program is engaged by insults of markedly different origin.

Accordingly, this review is organized to follow the biology rather than the literature. We first outline the core molecular machinery of ferroptosis; we then trace how mastitis-associated triggers engage each of its three pillars in turn—autophagic iron liberation, lipid-metabolic remodeling that expands the peroxidation-susceptible substrate pool, and collapse of the Nrf2-controlled system Xc^−^/glutathione/GPX4 axis—before evaluating the therapeutic strategies that restore this axis and the ferroptosis-associated genes that may serve as breeding markers. Throughout, individual studies are stratified by evidentiary strength, distinguishing those providing direct ferroptosis evidence from those inferring involvement through upstream omics signatures alone, and we close by examining the compartment-specific consequences of ferroptosis inhibition and the unresolved questions that follow from them. By integrating mechanistic insight with emerging interventional strategies, this review aims to define the conditions under which ferroptosis becomes a tractable target for the prevention and treatment of mastitis.

## 2. Methodology: Literature Identification and Evidence Appraisal

This is a narrative review, and the literature was assembled through a structured but non-systematic search rather than a PRISMA-type protocol. PubMed, Web of Science, Scopus and Google Scholar were searched using combinations of the terms “ferroptosis,” “ferritinophagy,” “mitophagy,” “chaperone-mediated autophagy,” “GPX4,” “NCOA4,” “Nrf2” and “iron,” each paired with “mastitis,” “mammary epithelial cell,” “intramammary infection” or the individual pathogen names (*Staphylococcus aureus*, *Escherichia coli*, *Klebsiella pneumoniae*, *Streptococcus uberis*, Group B *Streptococcus*). The search prioritized literature published between 2020 and 2026 to capture the most current mechanistic and translational work in this fast-moving field, and was extended back to 2014 where an older source was needed to establish a foundational concept, such as the original biochemical characterization of GPX4-regulated ferroptosis. Reference lists of retrieved articles and prior reviews were screened manually to identify additional sources this search strategy would otherwise have missed.

Peer-reviewed original studies and reviews reporting ferroptosis-related mechanisms, readouts or interventions in mammary tissue or in directly relevant comparator systems were eligible for inclusion. Conference abstracts and non-peer-reviewed preprints were excluded. Because the underlying literature is drawn from heterogeneous bovine, caprine, murine and in vitro systems that differ in how directly they demonstrate ferroptosis, each cited study was additionally graded on the strength of its ferroptosis evidence using a three-tier scheme. Studies graded as direct report lipid peroxidation or iron-flux readouts together with rescue by a ferroptosis inhibitor such as ferrostatin-1 or deferiprone, or by genetic loss-of-function. Studies graded as partial report a biochemical signature consistent with ferroptosis but without a ferroptosis-specific pharmacological or genetic control. Studies graded as inferred report only upstream transcriptomic or metabolomic signatures, without measurement of execution-stage markers such as lipid peroxidation or labile iron. This grading is carried out through the interpretive discussion throughout the review, so that claims resting on direct mechanistic evidence are distinguished from those resting on inference.

## 3. The Molecular Machinery of Ferroptosis

Several metabolic programs set how readily a cell reaches this point. Redox homeostasis, iron metabolism, mitochondrial activity, and amino acid, lipid, and sugar metabolism all feed in, which is why susceptibility varies so widely between cell types and conditions [25]. Cells that have died this way share a recognizable profile: mitochondrial atrophy, iron accumulation, lipid peroxidation, and reduced glutathione (GSH) content and GPX4 expression [26]. The shrunken mitochondrion is the most distinctive of these and is what separates ferroptosis from apoptosis under electron microscopy.

### 3.1. The System Xc^−^/GSH/GPX4 Antioxidant Axis

Ferroptotic fate ultimately depends on the cell’s capacity to neutralize lipid hydroperoxides before they propagate. Central to this defense are the cystine/glutamate antiporter system Xc− (SLC7A11), which imports cystine for GSH synthesis, and GPX4, which uses GSH to reduce phospholipid hydroperoxides to inert alcohols. Both are transcriptionally controlled by nuclear factor erythroid 2-related factor 2 (Nrf2), which also regulates heme oxygenase-1 (HO-1) and other cytoprotective genes. As the most abundant endogenous antioxidant, GSH serves as the essential cofactor for GPX4 in neutralizing ROS [27]. GPX4 is a critical regulator of ferroptosis, and knockout of the GPX4 gene induces ferroptosis in most cells [28]. GSH synthesis is associated with SLC7A11 levels, and the SLC7A11 protein indirectly promotes GSH production. Emerging research has consistently demonstrated that the dynamic equilibrium in the initiation and progression of ferroptosis is linked to oxidative stress mechanisms [29,30,31].

### 3.2. Iron Handling, Ferritinophagy and the Labile Iron Pool

Iron homeostasis is maintained by ferritin heavy chain 1 (FTH1) and ferritin light chain (FTL), which function as iron-storage proteins by oxidizing Fe^2+^ to Fe^3+^, thereby sequestering intracellular labile iron and limiting Fenton-mediated oxidative damage. Ferritinophagy—NCOA4 (nuclear receptor coactivator 4)-mediated selective autophagy of ferritin—represents the principal source of the catalytic iron flux that drives ferroptosis. NCOA4 acts as a cargo receptor that binds FTH1, delivering ferritin to autophagosomes for lysosomal degradation and release of ferrous ions [32]. The liberated iron then fuels lipid peroxidation and depletes GSH, driving GPX4 inactivation and ferroptosis [33]. Pathological stresses can upregulate this pathway; for example, the *Aeromonas hydrophila* effector Hcp1 can induce upregulation of NCOA4, thereby depleting cytoprotective ferritin iron stores in splenic macrophages and causing the accumulation of intracellular Fe^2+^ [34]. NCOA4-mediated ferritinophagy is therefore a key driver of ferroptosis through the liberation of iron.

### 3.3. Lipid Substrate, the Role of ACSL4, and Selenoprotein Defense

**Figure 1:**
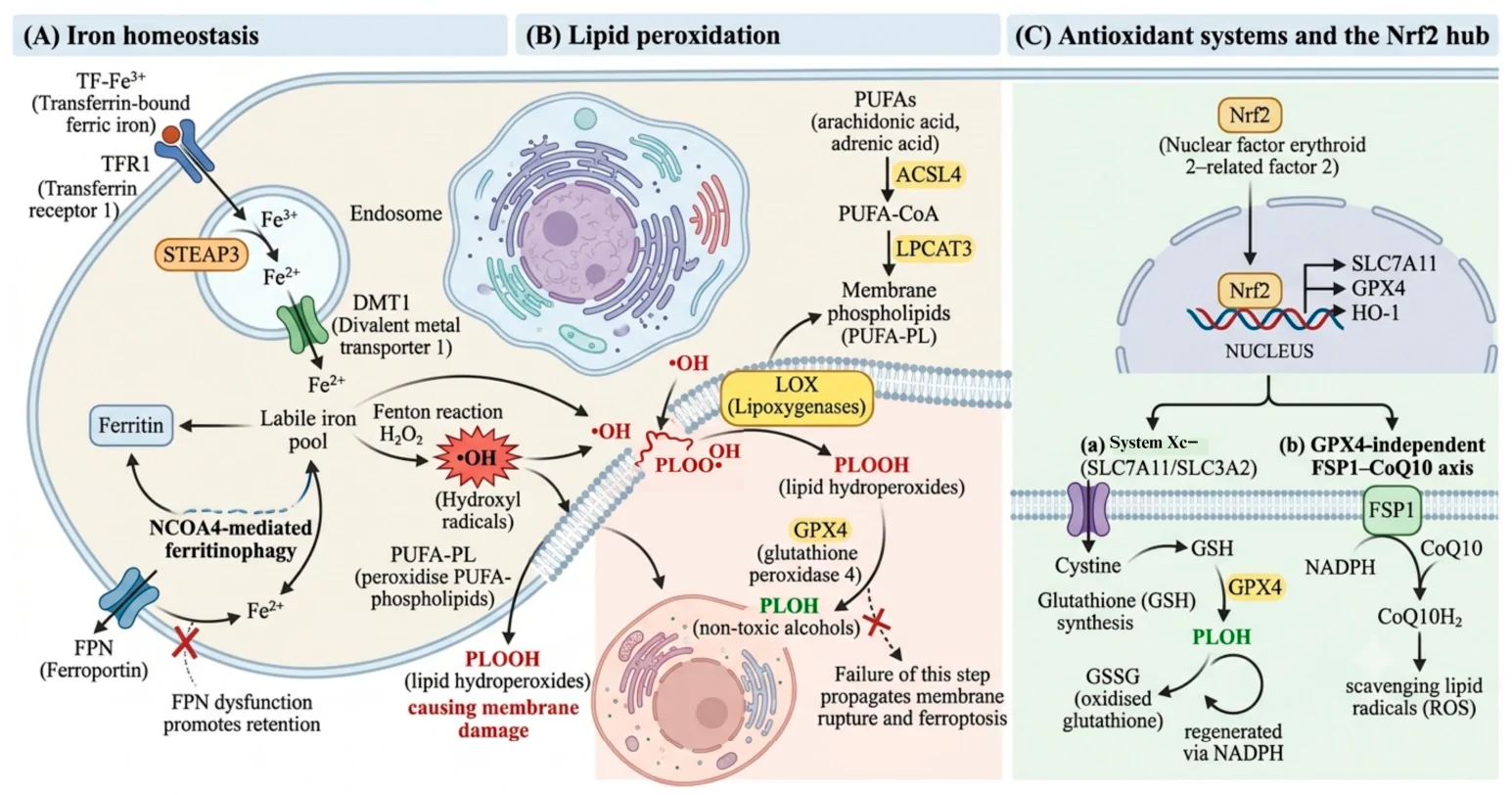
Molecular pathways of ferroptosis in mammary epithelial cells during mastitis. The figure illustrates the three interdependent biochemical pillars that drive ferroptotic cell death: (**A**) Iron homeostasis and liberation via NCOA4-mediated ferritinophagy, showing transferrin-bound iron uptake through TFR1, STEAP3-mediated Fe^3+^→Fe^2+^ conversion, DMT1-mediated import into the labile iron pool, and ferroportin (FPN)-mediated export (dysfunction of which promotes iron retention). (**B**) Lipid peroxidation cascade, depicting ACSL4-mediated esterification of PUFAs (arachidonic acid) into membrane phospholipids, LOX-catalyzed peroxidation to lipid hydroperoxides (PLOOH), and GPX4-dependent detoxification to non-toxic lipid alcohols using glutathione (GSH). (**C**) Antioxidant systems centered on the Nrf2 transcriptional hub, which drives expression of SLC7A11 (System Xc^−^ subunit), GPX4, and HO-1, alongside the GPX4-independent FSP1–CoQ10 axis that provides additional ferroptosis resistance. The convergence of iron overload, accumulation of lipid-peroxidation substrates, and antioxidant failure triggers membrane rupture and ferroptotic cell death. Created in BioRender (https://BioRender.com/gtv2jsl (accessed on 13 September 2026)).

Iron and antioxidant status determine whether peroxidation proceeds, but the reaction itself requires PUFA-esterified phospholipid substrate. The susceptibility of a membrane to ferroptosis is therefore governed in large part by its lipid composition, which is set by acyl-CoA synthetase long-chain family member 4 (ACSL4)-mediated esterification of PUFAs—particularly arachidonic acid (AA)—into membrane phospholipids, followed by their iron-dependent peroxidation (Figure 1). Beyond the canonical GPX4 system, selenium-dependent enzymology provides an additional regulatory layer. Peroxiredoxin 6 (PRDX6) specifically transports the reactive metabolic intermediate selenium by forming a persulfide bond at the C47 residue [35]; furthermore, PRDX6 enhances selenium utilization for selenoprotein synthesis through complex formation with selenophosphate synthetase 2 (SEPHS2), thereby directly promoting GPX4 expression and thereby counteracting ferroptosis [36]. These mechanistic pillars—iron, lipid substrate and antioxidant capacity—provide the conceptual scaffold against which pathogen-driven ferroptosis in mastitis can be understood.

## 4. Ferroptosis as a Converging Mode of Epithelial Death in Mastitis

A growing body of evidence indicates that the principal pathogens of mastitis can engage all three biochemical pillars of ferroptosis, supporting a role for it as a prominent contributor to mammary epithelial injury rather than a merely incidental consequence of infection. Although the supporting studies come from different pathogens and model systems, ferroptosis emerges as a recurring—and plausibly shared—endpoint across phylogenetically distinct mastitis pathogens. In both murine mastitis models and primary mammary epithelial cells, *Staphylococcus aureus* induces a dose-dependent increase in cell death accompanied by accumulation of malondialdehyde (MDA, the terminal product of PUFA peroxidation) and depletion of GSH—the canonical biochemical signature of ferroptosis [21]. The causal nature of this relationship is established pharmacologically: the radical-trapping antioxidant ferrostatin-1 (Fer-1), which directly interrupts lipid peroxidation, and the iron chelator deferiprone (DFO), which removes the catalytic iron required for the Fenton reaction, both significantly attenuate *S. aureus*-induced death in mammary epithelial cells [21]. An analogous ferroptotic program is triggered by the Gram-negative pathogen *Escherichia coli* in bovine mammary epithelial cells, demonstrating that ferroptosis is not restricted to Gram-positive infection but represents a conserved route of epithelial demise in mastitis [37]. The breadth of triggers and the recurring molecular signatures across these studies are summarized in Table 1.

Direct ferroptosis evidence classifies each study by the strength of its mechanistic demonstration: Direct = lipid peroxidation/iron readouts plus rescue by a ferroptosis inhibitor (e.g., Fer-1/DFO) or genetic loss-of-function; Partial = consistent biochemical signature without a ferroptosis-specific control; Inferred = ferroptosis implied from omics signatures without measurement of execution-stage markers. Abbreviations: MMECs, mouse mammary epithelial cells; bMECs, bovine mammary epithelial cells; GMECs, goat mammary epithelial cells; KD, knockdown; LIP, labile iron pool; 4-HNE, 4-hydroxynonenal; Fer-1, ferrostatin-1; DFO, deferiprone; CMA, chaperone-mediated autophagy; AA, arachidonic acid. Arrows indicate changes in expression or levels: ↑ denotes upregulation/increased levels; ↓ denotes downregulation/decreased levels.

## 5. Autophagy-Mediated Iron Liberation as a Unifying Mechanism

**Figure 2:**
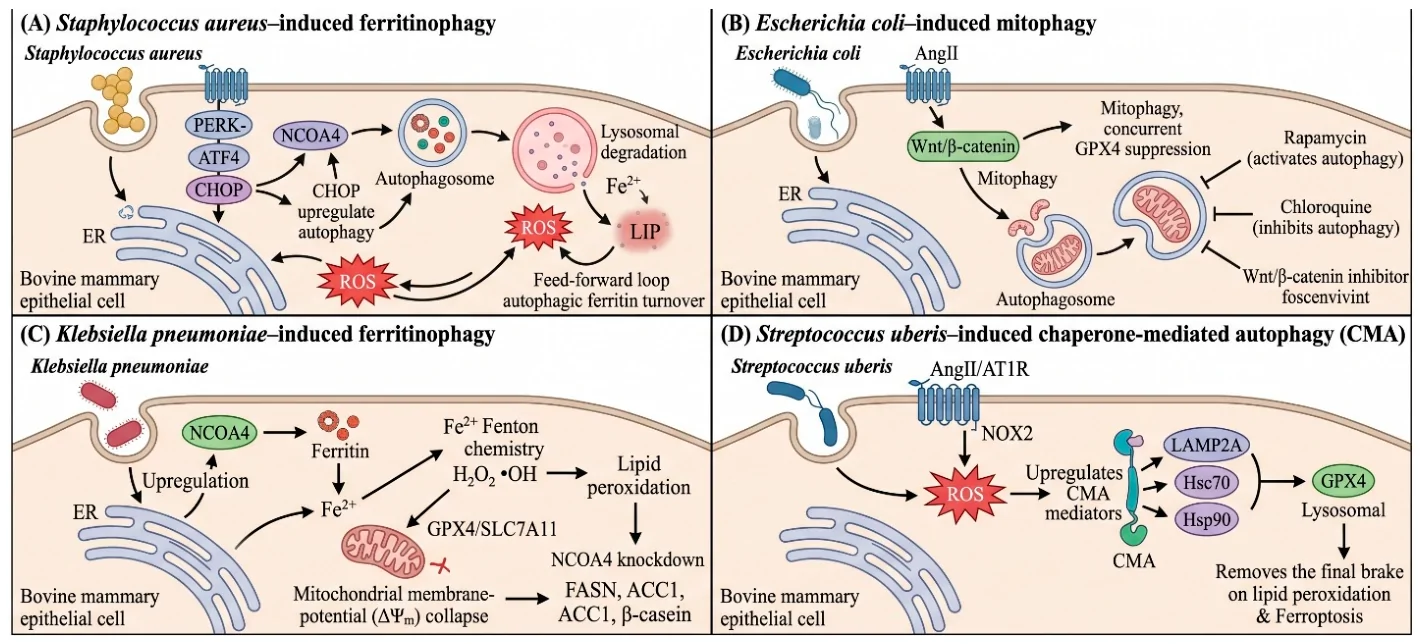
Autophagy-mediated iron liberation as a unifying upstream mechanism across mastitis pathogens. The figure depicts the three complementary autophagic programs through which phylogenetically distinct mastitis pathogens mobilize sequestered iron to fuel ferroptotic lipid peroxidation. (**A**) *Staphylococcus aureus*-induced ferritinophagy: infection activates the PERK–eIF2α–ATF4–CHOP branch of the unfolded protein response, which upregulates autophagy and promotes NCOA4-mediated delivery of ferritin to autophagosomes for lysosomal degradation, releasing Fe^2+^ into the labile iron pool; a feed-forward loop operates whereby the resulting ROS further amplify ER stress, sustaining autophagic ferritin turnover. (**B**) *Escherichia coli*-induced mitophagy: infection activates the Wnt/β-catenin pathway, which promotes mitophagy and concurrent GPX4 suppression; the autophagic dependence of this process is confirmed by rapamycin-mediated potentiation of ferroptosis and by chloroquine- and the Wnt/β-catenin inhibitor foscenvivint-mediated suppression. (**C**) *Klebsiella pneumoniae*-induced ferritinophagy: infection upregulates NCOA4, driving ferritin degradation and Fe^2+^ accumulation, which trigger Fenton chemistry-driven lipid peroxidation, mitochondrial membrane potential collapse, and suppression of the GPX4/SLC7A11 antioxidant axis; NCOA4 knockdown reverses these effects and partially restores lactation markers (FASN, ACC1, β-casein). (**D**) *Streptococcus uberis*-induced chaperone-mediated autophagy (CMA): infection activates the AngII/AT1R/NOX2 pathway, generating ROS that upregulate the CMA mediators LAMP2A, Hsc70, and Hsp90, which target GPX4 for lysosomal degradation, thereby removing the final brake on lipid peroxidation. Despite distinct upstream triggers, all four pathogens converge on the autophagic degradation of iron-storage or iron-containing structures to expand the catalytic iron pool required for ferroptosis. Created in BioRender (https://BioRender.com/1ika1ke (accessed on 13 September 2026)).

A key mechanistic question is how infection generates the labile iron pool that catalyzes lipid peroxidation. Two complementary lines of evidence converge on autophagy as the proximal mechanism that liberates sequestered iron during mastitis (Figure 2). In *S. aureus*-infected mammary epithelial cells, infection activates the protein kinase R-like endoplasmic reticulum kinase (PERK)–eukaryotic initiation factor 2α (eIF2α)-activating transcription factor 4 (ATF4)–C/EBP homologous protein (CHOP) branch of the unfolded protein response; this endoplasmic reticulum (ER) stress signal in turn upregulates autophagy, which degrades ferritin and thereby releases stored iron in a form competent to drive ferroptosis [21]. Because ferritin is the principal intracellular iron-storage protein, its autophagic turnover—ferritinophagy, typically mediated by the cargo receptor NCOA4—directly couples the autophagic machinery to expansion of the redox-active iron pool. This axis operates as a feed-forward loop: excess ROS not only drive lipid peroxidation but also trigger ER stress, activating the p-PERK–p-eIF2α–ATF4–CHOP signaling cascade, which reinforces the autophagy–iron–peroxidation circuit [21].

A parallel iron-liberating mechanism operates through the mitochondrion. Iron overload damages mitochondria and activates mitophagy, both of which can trigger and amplify ferroptosis. In *E. coli*-infected bovine mammary epithelial cells, infection activates the Wnt/β-catenin pathway, which promotes mitophagy and suppresses GPX4 expression, thereby driving ferroptosis [37]. The autophagic dependence of this process is confirmed pharmacologically: the autophagy/mitophagy inducer rapamycin potentiates ferroptosis, whereas the autophagy inhibitor chloroquine suppresses it, and the Wnt/β-catenin inhibitor foscenvivint attenuates the response [37]. Considered together, these studies reveal a unifying principle: although the upstream triggers differ—ER-stress-driven ferritinophagy following *S. aureus* infection versus Wnt/β-catenin-driven mitophagy following *E. coli* infection—both pathogens exploit autophagic degradation of iron-containing structures to generate the catalytic iron required for ferroptotic lipid peroxidation.

This ferritinophagy paradigm extends robustly to Gram-negative *Klebsiella pneumoniae* mastitis, a severe and therapeutically intractable threat to livestock. *K. pneumoniae* infection triggers iron accumulation in bovine mammary epithelial cells, which serves as a catalyst driving the lipid peroxidation cascade and ferroptosis [15,53]. Mechanistically, infection activates NCOA4, promoting ferritin degradation and elevating intracellular Fe^2+^, thereby triggering Fenton chemistry-driven lipid peroxidation, mitochondrial membrane potential collapse, and GPX4/SLC7A11 suppression—hallmark ferroptotic events [38]. RNA sequencing confirmed enrichment in ferroptosis, autophagy, and iron-metabolism pathways, and NCOA4 knockdown reversed these effects by restoring GPX4/SLC7A11 expression, reducing bacterial load, alleviating lipid peroxidation, and partially rescuing lactation markers (FASN, ACC1, β-casein). In vivo, infected mammary glands exhibited iron overload, 4-HNE accumulation, and NCOA4/FTH1 upregulation that correlated with the severity of pathological damage and lactation suppression. These findings identify ferritinophagy as a potential therapeutic target for preserving mammary function during bacterial mastitis [38].

## 6. Lipid Metabolic Remodeling Expands the Ferroptosis Substrate Pool

Iron and antioxidant status determine whether peroxidation proceeds, but the reaction requires a PUFA-esterified phospholipid substrate, and several observations indicate that mastitis is accompanied by lipid metabolic reprogramming that biases the mammary epithelial membrane toward a peroxidation-susceptible state. Lipid profiling of mammary glands from dairy cows with clinical mastitis reveals significant upregulation of ACOT7, an enzyme that influences long-chain fatty acid handling and unsaturated fatty acid biosynthesis; by enlarging the cellular pool of PUFA-containing lipids, ACOT7 may increase substrate availability for ACSL4-driven esterification and subsequent peroxidation, thereby sensitizing epithelial cells to ferroptosis [51]. Consistent with this, multi-omic analysis along the gut–metabolism–mammary axis identifies arachidonic acid metabolism as a principal pathway altered in mastitis, with elevated AA metabolites in mammary tissue [52]. Because AA is a preferred substrate for ACSL4-mediated phospholipid incorporation and a primary fuel for ferroptotic peroxidation, this enrichment provides a metabolic substrate for the execution step of ferroptosis. The same study links mastitis to disruption of the blood–milk barrier, downregulation of extracellular matrix–receptor and focal adhesion pathways, and gut dysbiosis (Proteobacteria enrichment) with altered bile acid and amino acid profiles—changes concordant with ferroptosis-associated epithelial loss and cytoskeletal reorganization [52]. It should be stressed, however, that these transcriptomic and metabolomic shifts (for example changes in ACOT7, arachidonic acid metabolites or BLV-associated markers) describe an expansion of the peroxidation-susceptible substrate pool and are consistent with—but do not by themselves demonstrate—ferroptosis execution, since lipid peroxidation, labile iron flux and rescue by ferrostatin-1 were not directly measured in these datasets.

A complementary mechanism links innate immunity directly to the supply of lipogenic substrates. STING activation in *S. aureus*-infected mammary epithelial cells drive ferroptosis via lipid-metabolic reprogramming: STING interacts with FASN to promote de novo fatty acid synthesis, thereby providing substrates for SCD2 and ACSL4 (key ferroptosis-executing enzymes) to generate lipid peroxides [25]. Concurrent GSH depletion and ROS accumulation create a pro-ferroptotic environment. Genetic or pharmacological STING inhibition reverses these hallmarks and restores redox balance, indicating that the STING–FASN–ACSL4 axis constitutes a ferroptosis pathway linking innate immunity to metabolic dysregulation in mastitis [25]. Several of these lipidomic and proteomic associations were not accompanied by direct measurements of ferroptosis markers (GPX4, lipid peroxidation, iron accumulation); nonetheless, they outline a mechanistically coherent route by which altered fatty acid metabolism could enable ferroptotic membrane damage in the inflamed gland [51,52].

## 7. Collapse of the GPX4/Nrf2 Antioxidant Axis and Its Therapeutic Restoration

**Figure 3:**
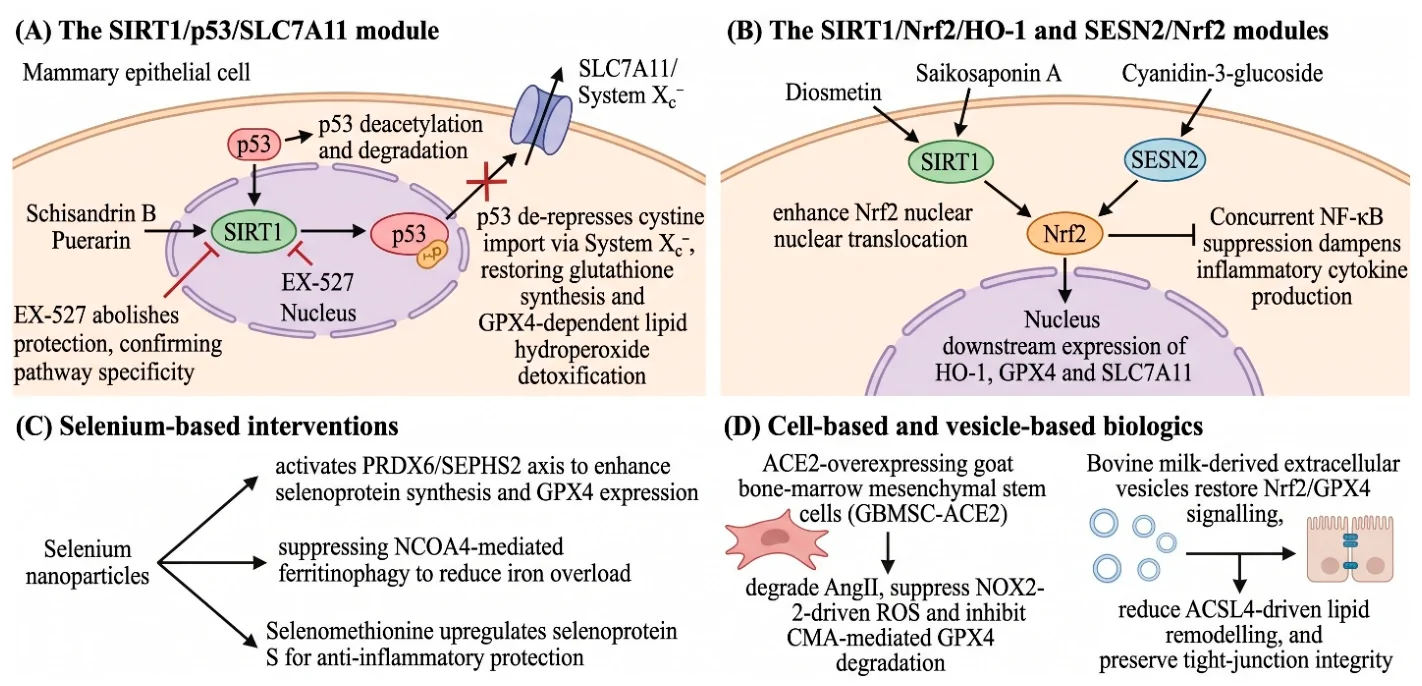
Pharmacological and biological strategies to restore the antioxidant axis and suppress ferroptosis in mastitis. The figure summarizes the principal molecular targets through which therapeutic, nutritional and cell-based interventions counteract ferroptotic mammary epithelial injury. (**A**) The SIRT1/p53/SLC7A11 module: schisandrin B and puerarin upregulate SIRT1, which promotes p53 deacetylation and degradation. Because p53 transcriptionally represses SLC7A11, its removal derepresses cystine import via System Xc^−^, restoring glutathione (GSH) synthesis and GPX4-dependent lipid hydroperoxide detoxification. The SIRT1 inhibitor EX-527 abolishes the protective effect, confirming pathway specificity. (**B**) The SIRT1/Nrf2/HO-1 and SESN2/Nrf2 modules: diosmetin and saikosaponin A activate SIRT1 to enhance Nrf2 nuclear translocation and downstream expression of HO-1, GPX4 and SLC7A11, while cyanidin-3-glucoside activates SESN2 upstream of Nrf2; concurrent NF-κB suppression dampens inflammatory cytokine production. (**C**) Selenium-based interventions: selenium nanoparticles activate the PRDX6/SEPHS2 axis to enhance selenoprotein synthesis and GPX4 expression, while simultaneously suppressing NCOA4-mediated ferritinophagy to reduce iron overload; selenomethionine upregulates selenoprotein S for anti-inflammatory protection. (**D**) Cell-based and vesicle-based biologics: ACE2-overexpressing goat bone marrow mesenchymal stem cells (GBMSC-ACE2) degrade AngII, suppress NOX2-driven ROS, and inhibit CMA-mediated GPX4 degradation; bovine milk-derived extracellular vesicles restore Nrf2/GPX4 signaling, reduce ACSL4-driven lipid remodeling, and preserve tight junction integrity. Arrows indicate activation (→) or inhibition (⊣); dashed lines denote indirect or multi-step connections. Created in BioRender (https://BioRender.com/lb42dza (accessed on 13 September 2026)).

Mastitis is characterized by a collapse of the antioxidant axis, which serves as the final brake on lipid hydroperoxide accumulation [27]. Across *S. aureus* infection models, Fe^2+^ and MDA levels rise while GSH, GPX4, and ferritin levels fall, indicating simultaneous iron loading, antioxidant depletion, and loss of hydroperoxide-detoxifying capacity [54]. This convergence identifies the SLC7A11/GPX4 node, together with its upstream regulators, as the logical target for therapeutic intervention. Consistent with this framework, a series of naturally derived compounds confer protection in *S. aureus* mastitis by restoring the antioxidant arm while simultaneously dampening inflammation, and their shared mechanistic logic reinforces the centrality of ferroptosis to disease (Figure 3, Table 2).

### 7.1. The SIRT1/p53/SLC7A11 Module

Schisandrin B, the principal derivative of *Schisandra chinensis*, attenuates *S. aureus*-induced ferroptosis by upregulating SIRT1 and SLC7A11, downregulating p53, and suppressing NF-κB activation. Because p53 transcriptionally represses SLC7A11, SIRT1-dependent reduction in p53 de-represses cystine uptake and restores GSH-dependent GPX4 activity, lowering Fe^2+^ and MDA while preserving GPX4 and ferritin, together with reduced IL-1β, TNF-α, and myeloperoxidase (MPO) activity [54]. A parallel mechanism operates for puerarin, which alleviates *S. aureus*-induced mastitis by activating SIRT1/p53/SLC7A11 to suppress ferroptosis and inflammation: puerarin restored GPX4 and GSH, reduced iron overload, lipid peroxidation, and inflammatory cytokines, and its protection was abolished by the SIRT1 inhibitor EX-527, confirming that these effects are SIRT1-dependent [55].

### 7.2. The SIRT1/Nrf2 and SESN2/Nrf2 Modules

Two additional compounds converge on the SIRT1/Nrf2 axis. Diosmetin and saikosaponin A each upregulate SIRT1 to enhance Nrf2 nuclear translocation and downstream HO-1/GPX4 expression, reversing *S. aureus*-induced iron accumulation, GPX4 suppression, and antioxidant depletion, while inhibiting NF-κB-driven inflammation and preserving blood–milk barrier integrity. In both cases, the SIRT1 inhibitor EX-527 abolished protection, confirming pathway specificity [56,57]. Cyanidin-3-glucoside (C3G) extends this logic upstream, activating SESN2 to drive Nrf2-dependent GPX4 and SLC7A11 expression and thereby reducing oxidative stress, lipid peroxidation and ferroptotic death; concurrently, C3G exerts antimicrobial effects against *S. aureus* and MRSA while downregulating IL-1β, IL-6 and TNF-α and inhibiting STAT2/STAT3 signaling, offering a comprehensive strategy that addresses infection, inflammation and cell death [58]. Gardenoside similarly inhibits ferroptosis by reducing MDA and iron content while restoring GSH and upregulating GPX4, SLC7A11 and ferritin through the Nrf2/HO-1 pathway, with concurrent upregulation of Nrf2, SLC7A11 and GPX4 confirming action through the Nrf2/SLC7A11/GPX4 axis [59].

### 7.3. Nrf2-Dependence Confirmed by Pharmacological Loss of Function

The dependence of mammary epithelial protection on Nrf2 is corroborated across several oxidative injury and infection models in which a selective Nrf2 inhibitor abolishes this protection. Baicalin suppresses H_2_O_2_-induced ROS accumulation, inflammatory cytokine production (TNF-α, IL-6, IL-1β), and cell death through Nrf2 activation. The Nrf2 inhibitor retinoic acid abolishes this protective effect. Although this study did not directly quantify ferroptosis markers, its Nrf2-dependent suppression of iron-catalyzed ROS and lipid peroxidation is mechanistically consistent with ferroptosis inhibition [60]. Forsythiaside A (FTA) alleviates LPS-induced mastitis by inhibiting ferroptosis via the GPX4/SLC7A11 axis, reducing ROS, lipid peroxidation, and iron while restoring SOD and T-AOC. The ferroptosis inducer RSL3 partially reverses this protection, and FTA additionally activates Nrf2 and suppresses NF-κB/NLRP3 [44]. Stigmasterol, a bioactive component of *Prunella vulgaris* L., exerts dual anti-inflammatory and anti-ferroptotic effects by suppressing TLR4/NF-κB and activating Nrf2/GPX4. The Nrf2 inhibitor ML385 abolishes its protection, and both the plant extract and purified stigmasterol attenuate tissue damage and restore barrier function in vivo [34]. Fisetin likewise alleviates LPS-induced mastitis by modulating oxidative stress and ferroptosis via the Nrf2/GPX4 axis, enhancing the xCT/GSH/GPX4 system, and reshaping the gut microbiota to increase short-chain fatty acid levels. Together, these effects combine ferroptosis suppression, antioxidant defense, and microbiome modulation into a potential antibiotic-alternative strategy [43].

This Nrf2/GPX4-centered logic also rationalizes the activity of broader classes of dietary polyphenols, whose anti-inflammatory effects in mammary tissue are increasingly attributed, at least in part, to modulation of ferroptosis. Curcumin, schisandrin B, caffeic acid and chlorogenic acid have each been highlighted as natural bioactive compounds that alleviate mammary inflammation by acting on ferroptotic signaling [14,23,24]: curcumin and caffeic acid are well-characterized Nrf2 inducers and radical scavengers whose capacity to restore the SLC7A11/GSH/GPX4 axis and quench lipid peroxidation positions them within the same mechanistic family as the validated agents above, even where mammary-specific ferroptosis end-points remain to be measured directly. These findings indicate that the anti-ferroptotic pharmacology of mastitis is converging on a small number of master regulatory nodes, chiefly Nrf2, SIRT1, GPX4, and SLC7A11, rather than on compound-specific mechanisms. This convergence both simplifies rational drug selection and supports head-to-head comparisons of efficacy in naturally infected animals.

**Table 2:** Therapeutic, nutritional and cell-based modulators of ferroptosis in mastitis and their molecular targets.

Agent	Source/Class	Molecular Target/Axis	Ferroptosis-Relevant Outcome	References
Schisandrin B	*Schisandra chinensis*	SIRT1/p53/SLC7A11; NF-κB suppression	↓ Fe^2+^/MDA; ↑ GSH/GPX4/ferritin; ↓ IL-1β/TNF-α/MPO	[54]
Puerarin	Isoflavone (*Pueraria*)	SIRT1/p53/SLC7A11	Restores GPX4/GSH; EX-527 abolishes protection	[55]
Diosmetin	Flavonoid	SIRT1/Nrf2/HO-1/GPX4	↓ MPO/Fe^2+^/MDA; EX-527-sensitive	[56]
Saikosaponin A	*Bupleurum* saponin	SIRT1/Nrf2/HO-1/GPX4; NF-κB inhibition	Restores GPX4; preserves blood–milk barrier	[57]
Gardenoside	Iridoid glycoside	Nrf2/SLC7A11/GPX4	↓ MDA/iron; ↑ GSH/ferritin; ↓ NF-κB	[59]
Cyanidin-3-glucoside	Anthocyanin	SESN2/Nrf2; STAT2/STAT3 inhibition	↑ GPX4/SLC7A11; antimicrobial vs. MRSA	[58]
Quercetin	Flavonoid	TfR1/NCOA4 ↓; SLC7A11/GPX4 ↑; NLRP3 inhibition	Restores FTH1; preserves Occludin/ZO-1	[41]
Fisetin	Flavonoid	Nrf2/GPX4; xCT/GSH; microbiome (SCFAs)	↓ Fe^2+^/ROS; preserves barrier	[43]
Forsythiaside A	Phenylethanoid glycoside	GPX4/SLC7A11; Nrf2; NF-κB/NLRP3 inhibition	RSL3-reversible; ↑ SOD/T-AOC	[44]
Stigmasterol	*Prunella vulgaris* phytosterol	Nrf2/GPX4; TLR4/NF-κB; MLCK	ML385-sensitive; preserves tight junctions	[34]
Baicalin	Flavone (*Scutellaria*)	Nrf2 activation (HO-1/NQO-1)	↓ H_2_O_2_-ROS, cytokines; RA abolishes	[60]
Chlorogenic acid	Phenolic acid	TLR4/CD36/FAK/mTORC1; ↓ IL-6	Prevents ferroptotic loss; preserves milk synthesis	[22]
Methionine	Sulfur amino acid	Nrf2; GPX4/SLC7A11/FTH1	↓ LIP/lipid-ROS under heat stress	[46]
l-Glutamic acid	Amino acid	Sirt5/Nrf2/Prdx1; mitochondrial fusion	Reduces lipid hydroperoxides; Sirt5-dependent	[61]
Myo-inositol	Carbocyclic polyol	Sirt5/Nrf2; GPX4/SLC7A11; OPA1/MFN	Restores mitochondrial fusion; ↓ MDA	[62]
Pulsatilla saponin B4	Triterpenoid saponin	AMPK/Nrf2/GSH-Px; SLC7A11/GPX4	↓ MDA/ROS; combined anti-ferroptotic and anti-apoptotic action	[63]
Selenium nanoparticles	Nanoselenium	PRDX6-SEPHS2/NRF2/GPX4; ↓NCOA4 ferritinophagy	↓ Iron overload and lipid peroxidation; improved hemodynamics	[64]
Selenomethionine	Selenoamino acid	Selenoprotein S upregulation	Anti-inflammatory effects in bMECs and macrophages	[65,66]
Milk-derived EVs	Extracellular vesicles	Nrf2/GPX4; ACSL4; S100A4	Restores barrier integrity; ↓ ROS relative to *K. pneumoniae* infection	[53]
GBMSC-ACE2	Engineered stem cells	ACE2/AngII/NOX2-CMA-GPX4	↓ SCC and bacterial load; stabilized GPX4	[39]
Ferrostatin-1/Deferiprone	Reference inhibitors	Radical trapping/iron chelation	Blocks lipid peroxidation; removes iron	[15,21]

Arrows denote the direction of change produced by the agent (↑ denotes upregulation/increased levels; ↓ denotes downregulation/decreased levels). EVs, extracellular vesicles; GBMSC-ACE2, ACE2-overexpressing goat bone-marrow mesenchymal stem cells; SCFAs, short-chain fatty acids; MRSA, methicillin-resistant *Staphylococcus aureus*.

## 8. Inflammatory Mediators, GSH Metabolism and Mitochondrial Dynamics

### 8.1. IL-6 as an Inflammatory Driver of Ferroptosis

Pro-inflammatory cytokine signaling is increasingly recognized as an upstream initiator of ferroptosis in the mammary gland. LPS induces ferroptosis in goat mammary epithelial cells via USP14-mediated deubiquitination and stabilization of IL-6, which promotes ferroptosis through NRF2-pathway suppression: LPS upregulated IL-6 protein by reducing its ubiquitination, leading to increased Fe^2+^ accumulation, ROS elevation, GSH depletion, MDA production and TNF-α secretion, all reversed by ferrostatin-1, while the NRF2 inhibitor brusatol reversed the protection conferred by IL-6 silencing—thereby establishing the USP14/IL-6/NRF2 ferroptosis axis as a source of novel therapeutic targets [42]. Complementing this, LPS orchestrates IL-6 release via the TLR4/CD36/FAK/mTORC1 axis, triggering ferroptosis and impairing milk synthesis in bovine mammary epithelial cells; chlorogenic acid (CGA) suppressed this cascade by inhibiting TLR4/CD36/FAK/mTORC1 signaling, reducing IL-6 release and preventing ferroptotic death, thereby preserving epithelial function and milk synthesis and linking inflammatory signaling directly to ferroptosis execution [22].

### 8.2. Glutathione-Metabolism Dysregulation as a Ferroptosis Checkpoint

A multi-omics study demonstrates that clinical mastitis drives ferroptosis through dysregulation of GSH metabolism: in affected tissues, GSTM3 and GSS downregulation led to GSH depletion (0.66-fold), ROS elevation (2.1-fold), and MDA accumulation (2.3-fold)—classic ferroptotic hallmarks. Because GSH is the essential cofactor for GPX4, its depletion sensitizes cells to lipid peroxidation; GSTM3 overexpression restored GSH, reduced ROS/MDA, and reversed these signatures, confirming the GSTM3–GSH axis as a critical checkpoint, while LPS-induced GSTM3/GSS suppression exacerbated oxidative damage in vivo, positioning the GSTM3–GSH–GPX4 axis as a promising therapeutic target [67].

### 8.3. Mitochondrial Dynamics, Amino Acids and the Sirt5/Nrf2 Axis

Mitochondrial integrity is a key determinant of ferroptotic susceptibility, and several nutritional interventions exploit this dependency. Myo-inositol (MI) alleviates oxidative stress in high-yielding dairy cows by activating the Sirt5/Nrf2 pathway: high-yielders exhibited redox imbalance, mitochondrial fission and MI depletion, and MI supplementation restored mitochondrial fusion (OPA1, MFN1, MFN2), reduced fission (Drp1, FIS1) and decreased MDA, with MI upregulating Sirt5 to enhance Nrf2-driven GPX4 and SLC7A11; Nrf2 inhibition abolished protection, confirming specificity [62]. In parallel, l-glutamic acid (l-Glu) alleviates LPS-induced mastitis by activating the Sirt5/Nrf2/Prdx1 axis, promoting Nrf2 nuclear translocation and upregulating Prdx1—a peroxidase that directly reduces lipid hydroperoxides—while restoring mitochondrial membrane potential and promoting mitochondrial fusion; the effects were abolished by Sirt5 knockdown or Nrf2 inhibition, positioning l-Glu as a ferroptosis-suppressive nutritional strategy [61]. Methionine (Met) likewise alleviates heat stress (HS)-induced ferroptosis in bovine mammary epithelial cells by activating Nrf2 to restore iron homeostasis and antioxidant defenses: HS elevated the labile iron pool, lipid ROS, MDA and mitochondrial dysfunction while reducing GPX4, SLC7A11 and FTH1, and Met reversed these effects, with FTH1 upregulation promoting safe iron sequestration and Nrf2 knockdown abolishing protection [46]. Pulsatilla saponin B4 (PSB4) protects bovine mammary epithelial cells from H_2_O_2_-induced damage via the AMPK/Nrf2/GSH-Px axis, enhancing SOD and GSH-Px activities while reducing MDA and ROS and concurrently reducing apoptosis, with Compound C confirming AMPK dependence and the suppression of GSH-Px decline indicating ferroptosis-specific protection [63].

## 9. Xenobiotic, Nanomaterial, Viral and Microbiota-Driven Ferroptosis

Ferroptosis in the mammary gland is not confined to classical bacterial infection but is also provoked by environmental and dietary insults that share the same execution machinery. Zinc oxide nanoparticles (ZnO-NPs) induce blood–milk barrier dysfunction in lactating mice by triggering ferroptosis alongside inflammation: ZnO-NP accumulation caused histopathological injury, disrupted tight-junction proteins and promoted FITC-albumin leakage, activating NF-κB and MAPK while driving iron accumulation and lipid oxidation, such that excessive iron overload fuels Fenton chemistry to compromise membrane integrity and tight-junction function [47]. Similarly, glyphosate (GLY) induces ferroptosis in MAC-T cells—characterized by Fe^2+^ accumulation, lipid peroxidation, ROS elevation and GSH depletion—triggering inflammatory responses and downregulating tight-junction proteins (ZO-1, Occludin, Claudin-3); ferroptosis served as the upstream driver of both inflammation and barrier dysfunction, as ferrostatin-1 significantly attenuated GLY-induced damage [48]. Pyocyanin (PCN) from *Pseudomonas aeruginosa* exacerbates inflammatory and oxidative injury in bovine mammary epithelial cells with features consistent with ferroptosis promotion: PCN increased MDA and ROS while depleting GSH-Px and T-AOC, downregulated Nrf2/HO-1 and activated p53 signaling (which can repress SLC7A11), with TLR4/NF-κB activation amplifying ROS and co-exposure to LPS intensifying all effects [42].

Viral and microbiota-derived insults complete this picture. Bovine leukemia virus (BLV), the cause of enzootic bovine leukosis, impairs mammary antimicrobial defense and aggravates clinical mastitis; in bovine mammary epithelial cells, wild-type BLV and a miRNA-deficient mutant elevated unsaturated fatty acids and disrupted vitamin B6 (VB6) metabolism, such that perturbation of the System Xc^−^ transporter (SLC7A11/SLC3A2) and VB6-dependent cysteine supply for γGC→GSH synthesis, together with elevated omega-3 (EPA/DPA/DHA) and omega-6 (arachidonic acid) PUFAs and lowered CoQ10, drives mammary-cell ferroptosis—possibly through BLV-encoded miRNAs [50]. At the level of the gastrointestinal–mammary axis, ruminal microbiota dysbiosis contributes to ruminant mastitis: a high-concentrate (70% grain) diet induced subacute ruminal acidosis in dairy goats, disrupting ruminal microbiota and raising serum LPS, TNF-α, IL-1β and milk somatic cell count, and transplanting the dysbiotic ruminal microbiota into mice triggered mastitis linked to oxidative stress and ferritinophagy–ferroptosis; mechanistically, elevated LPS upregulated transferrin receptor, promoted ferritinophagy and STEAP3-mediated Fe^3+^→Fe^2+^ conversion to drive Fenton-reaction ROS while depleting the System Xc^−^/GSH/GPX4 axis, with ferrostatin-1 alleviating mastitis [49].

## 10. Regulatory RNA, Selenium Biology and Advanced Therapeutics

Beyond metabolic and signaling control, post-transcriptional and biologic-based strategies are emerging. The circular RNA circ_HOMER3 exacerbates LPS-induced inflammatory injury in bovine mammary epithelial cells by coordinately activating the TLR4/MyD88/NF-κB and JAK1/STAT1 pathways while modulating ferroptosis via TNF/MAPK signaling. Overexpression promoted IκBα degradation and p65 nuclear translocation, upregulated IL-6, IL-8, and IL-1β, and reduced MAPK9 (JNK2) alongside downregulation of the ferroptosis regulator NCOA4. In contrast, silencing reduced Fe^2+^/Fe^3+^ accumulation and lipid-peroxidation markers while enhancing GSH and SOD. These findings position circ_HOMER3 as a molecular hub integrating inflammation, cell death, and ferroptosis [68].

Selenium-based nanomedicine has produced some of the most integrative results in the field. Selenium nanoparticles (SeNP) exhibit superior bioavailability over inorganic selenium [69,70], and function not only as antioxidants but also as regulators of iron metabolism [71]; they activate the NRF2 pathway, upregulating the selenoproteins GPX4 and PRDX6, which detoxify lipid hydroperoxides and thereby inhibit ferroptosis [72], and they can alleviate ferroptosis by upregulating X-box binding protein 1, which may suppress the NCOA4-ferritinophagy axis [73]. Building on the established role of NCOA4-mediated ferritinophagy in promoting ferroptosis during mastitis and the involvement of the PRDX6–GPX4 axis in counteracting it, oral administration of SeNP (20 μg/kg body weight) attenuated *K. pneumoniae*-induced mastitis in goats: the treatment reduced histopathological damage and neutrophil infiltration, restored anti-inflammatory IL-10 while suppressing IL-1β and IL-6, improved mammary hemodynamics and echotexture on Doppler ultrasonography, increased serum selenium and mammary glutathione, activated the PRDX6/SEPHS2 axis and upregulated GPX4, and crucially suppressed ferroptosis by inhibiting NCOA4-mediated ferritinophagy—reducing iron overload and lipid peroxidation and attenuating NCOA4–FTH1 colocalisation [64]. The broader rationale rests on the fact that persistent oxidative stress and iron dyshomeostasis exacerbate mammary epithelial injury [38,74], that selenomethionine inhibits inflammatory responses caused by *E. coli* via upregulation of selenoprotein S in bovine mammary epithelial cells and macrophages [65,66], and that ferroptosis has emerged as a critical pathogenic mechanism in inflammatory diseases including mastitis [58].

Two further biologic strategies address ferroptosis at the tissue level. Bovine milk-derived extracellular vesicles (EVs) alleviate *K. pneumoniae*-induced mastitis by suppressing ferroptosis and oxidative stress: infection downregulated GPX4, ACSL4 and S100A4 and reduced Nrf2/HO-1 signaling while increasing ROS and compromising tight junctions, and colostrum-derived, mature-milk and clinical-mastitis-milk EVs each reversed these effects, restoring GPX4 and Nrf2 expression, reducing ROS and preserving barrier integrity [53]. Finally, in *Streptococcus uberis* mastitis, infection drives ferroptosis in goat mammary epithelial cells via chaperone-mediated autophagy (CMA)-dependent GPX4 degradation: infection activates the AngII/AT1R/NOX2 pathway, increasing ROS and Fe^2+^ while upregulating the CMA mediators LAMP2A, Hsc70 and Hsp90 that promote GPX4 degradation and lipid peroxidation, and ACE2-overexpressing bone-marrow mesenchymal stem cells (GBMSC-ACE2) inhibit ADAM17, enhance ACE2, suppress NOX2-driven ROS and reduce CMA-mediated GPX4 loss, improving milk yield and reducing bacterial load and somatic cell count in vivo [39]. The same ACE2/AngII/NOX2–CMA–GPX4 ferroptosis axis was independently confirmed, with ACE2 stabilizing GPX4 by degrading AngII and attenuating NOX2 activity and with ferrostatin-1 phenocopying ACE2’s protective effects [40].

## 11. Ferroptosis-Associated Genes as Biomarkers for Mastitis-Resistance Breeding

The transcriptional footprint of ferroptosis offers a route to molecular diagnosis and genetic improvement. A proteomic study established that ferroptosis occurs in the mammary glands of Holstein cows with clinical mastitis, identifying *HMOX1* and *FTH1* as key players in iron homeostasis: elevated *HMOX1* releases pro-oxidant iron from heme to amplify the labile iron pool, while *FTH1* upregulation may represent a compensatory iron-sequestering response; both proteins localized to mammary epithelial cells and were upregulated in clinical-mastitis tissues and LPS-stimulated MAC-T cells, with erastin producing greater effects than LPS, confirming ferroptosis involvement [75]. A subsequent integrated transcriptomic and WGCNA analysis of MAC-T cells challenged with clinical *S. aureus* isolates identified ferroptosis, Toll-like receptor and TNF signaling as significantly enriched KEGG pathways and prioritized five iron-metabolism hub genes—*HMOX1*, *SLC11A2*, *STEAP3*, *SAT1* and *VDAC2*—with *HMOX1* and *SAT1* markedly upregulated and ferrostatin-1 reducing their expression, consistent with data from mother–daughter cow pairs with subclinical mastitis and highlighting these genes as candidates for molecular breeding [23].

Transcriptomic profiling of peripheral white blood cells from cows with subclinical mastitis revealed significant enrichment of the ferroptosis signaling pathway. Eight key genes (*SLC40A1*, *SLC11A2*, *FTH1*, *ACSL5*, *ACSL6*, *GCLC*, *CYBB*, and *VDAC3*) were identified as differentially expressed or alternatively spliced. *SLC40A1* (ferroportin) was upregulated, suggesting a compensatory response to iron overload, while *SLC11A2* (*DMT1*) mediated iron import. *FTH1* exhibited four alternative-splicing patterns, indicating complex post-transcriptional regulation of iron storage during inflammation. *ACSL5* and *ACSL6* may influence lipid-peroxidation substrate availability, *GCLC* is rate-limiting for GSH synthesis, *CYBB* (*NOX2*) generates superoxide, and *VDAC3* links mitochondrial dysfunction to ferroptotic susceptibility. The alternative splicing of *SLC40A1* and *FTH1* in particular offers potential biomarkers for genetic improvement of mastitis resilience [76]. In a related epigenetic study, DNA hypomethylation in *E. coli*-infected mastitis neutrophils upregulated SLC40A1 (ferroportin), the sole cellular iron exporter, whose dysregulation may increase labile iron pools that drive lipid peroxidation via Fenton chemistry; while ferroptosis in neutrophils may limit pathogen survival, excessive ferroptosis could exacerbate tissue damage, positioning SLC40A1 as an epigenetic target and implicating CITED2 and LGR4 as indirect modulators through HIF/GPX4 or Wnt/β-catenin pathways [77]. The role of ferroptosis has been extensively reviewed in relation to various veterinary diseases [14,78].

## 12. Bridging the Translational Gap: In Vitro Model Systems Versus In Vivo Intramammary Infection

A central caveat that qualifies the model proposed above is the distance between the systems in which ferroptosis has been demonstrated and the biology of natural mastitis. Much of the most direct mechanistic evidence derives from immortalized epithelial lines such as MAC-T [23,79], primary bovine, goat or mouse mammary epithelial cultures, or murine mastitis models challenged with a single pathogen, purified toxin (for example lipopolysaccharide or pyocyanin) or a defined agonist, frequently at high, non-physiological doses and over short timeframes [42,80]. These reductionist systems are indispensable for establishing causal chains and for pharmacological rescue experiments, but they cannot reproduce the multicellular architecture of the lactating gland, the dynamic recruitment of neutrophils and macrophages [81], the influence of milk components and the local iron environment [82,83], the polymicrobial and often chronic nature of field infection [84,85], or the substantial host-genetic variation among dairy animals [86]. Species specificity compounds this gap: several pillars of the proposed model rest on evidence from a single species—chaperone-mediated autophagy of GPX4, for instance, is documented in caprine mammary systems, whereas ferritinophagy and mitophagy data derive mainly from bovine and murine work—so the assumption that these routes co-occur within one host during a single natural infection remains to be tested. Consequently, findings obtained in isolated model systems should be regarded as mechanistically informative rather than directly generalizable, and the convergence described here is best framed as a set of pathway associations awaiting confirmation in naturally infected, genetically diverse herds using cell-type-resolved, execution-stage measurements.

## 13. Ferroptosis, Mitophagy and Autophagy in the Post-Vaccination Immune Response

A facet that has received little attention in the mastitis literature, yet carries clear translational relevance, is the influence of ferroptosis and the associated autophagic programs on the immune response elicited by vaccination. Beyond the mammary gland, redox-regulated cell death and autophagy are increasingly recognized as modulators of both innate priming and adaptive immunity: autophagy shapes antigen processing and presentation [87] and supports the survival and metabolic fitness of memory lymphocytes [88], while ferroptosis and iron flux influence dendritic-cell function, the polarization and persistence of effector cells [89], and the release of damage-associated molecular patterns that act as endogenous adjuvants [90]. In immunized mice, and in young adults receiving influenza vaccine, selenium supplementation increased GPX4 expression in T cells, expanded follicular helper T cell numbers and improved antibody responses [91]. Lipid peroxidation products and altered iron availability can therefore, in principle, either enhance or blunt the magnitude and durability of vaccine-induced protection, depending on their timing and cellular context [92,93]. For mastitis vaccines—whose field efficacy has historically been inconsistent [94,95,96]—these considerations suggest testable hypotheses: that the ferroptotic and autophagic status of mammary and infiltrating immune cells at the time of immunization may condition the quality of the local response, and that adjuvants or nutritional co-interventions modulating the Nrf2/GPX4/SLC7A11 axis (for example selenium supplementation) might be leveraged to improve immunogenicity. Direct experimental evidence linking these pathways to intramammary vaccine responses is at present largely absent, and dedicated studies—correlating ferroptosis and autophagy markers with antibody and cell-mediated readouts after vaccination, and testing pathway modulation as an adjuvant strategy—represent a promising and, to date, essentially unexplored avenue that could ultimately inform the design and scheduling of mastitis immunization programs.

## 14. From Mechanism to Practice: Implications for Udder-Health Preventive Schemes

The mechanistic picture assembled here has concrete implications for how clinicians and herd advisers might construct udder-health preventive schemes, provided the evidentiary caveats above are respected. First, because the ferritinophagy–ferroptosis cascade can be primed by lipopolysaccharide originating from ruminal and gut dysbiosis [3,97,98,99,100,101,102,103,104,105], dietary and management measures that stabilize the rumen—balanced concentrate-to-forage ratios, avoidance of subacute ruminal acidosis, and transition-period nutrition [12,13]—may pre-empt the upstream iron-liberation step before clinical disease develops, positioning nutrition as a first line of prevention rather than an adjunct. Second, the recurring dependence of protection on the Nrf2/GPX4/SLC7A11 axis identifies rational targets for nutritional supplementation: adequate selenium and antioxidant status [106,107,108], and bioactive feed additives with combined antimicrobial, anti-inflammatory and anti-ferroptotic activity (for example the phytochemicals cataloged in Table 2) [11], are plausible components of dry-off and peripartum prophylaxis, subject to evaluation of milk-residue and pharmacokinetic profiles. Third, ferroptosis-associated genes such as HMOX1, SAT1, SLC40A1 and FTH1, if validated, could be incorporated into genomic selection indices [20,23,76,77], allowing preventive schemes to operate at the level of resistance breeding as well as day-to-day management. Fourth, monitoring markers of iron dysregulation and lipid peroxidation [75] might in future help stratify cows by ferroptotic risk and time interventions to periods of greatest susceptibility, such as early lactation [106,108,109]. It must be emphasized that these recommendations are mechanism-guided rather than validated by controlled field trials; their role at present is to translate the biology into concrete, testable herd-level preventive strategies—integrating nutrition, targeted supplementation, and marker-assisted selection—that can be prospectively evaluated to assess their effect on clinical and subclinical mastitis incidence.

## 15. Synthesis: An Integrated Three-Pillar Model

Taken together, these studies motivate a proposed integrative model—depicted schematically in Figure 1—in which mastitis pathogens may initiate ferroptosis by engaging all three biochemical pillars of the pathway. Because the supporting evidence is drawn from different pathogens, host species and model systems, this model should be read as a hypothesis that associates the roles of ferritinophagy, mitophagy and chaperone-mediated autophagy in pathogen-induced ferroptosis rather than as an established universal paradigm. Infection-driven ROS and ER stress activate autophagic programs—ferritinophagy and mitophagy—that liberate catalytic iron [21,37]; concomitant remodeling of fatty acid metabolism expands the PUFA-phospholipid substrate available for peroxidation [51,52]; and depletion of the SLC7A11/GSH/GPX4 antioxidant axis removes the brake on lipid hydroperoxide accumulation [54]. The therapeutic exploitation of this model—through SIRT1/Nrf2/GPX4 activation, SIRT1/p53/SLC7A11 modulation, or Wnt/β-catenin inhibition—consistently limits both ferroptotic epithelial loss and inflammatory injury, underscoring the intimate crosstalk between ferroptosis and inflammation in the inflamed mammary gland [37,54,56,57,60]. A unifying theme across the natural-compound, nutritional, selenium-based and cell-based interventions cataloged in Table 2 is that the most effective agents act simultaneously on infection, inflammation and ferroptotic cell death—the three interconnected pathological pillars of mastitis—rather than on any single axis in isolation.

In weighing this body of work, it is important to recognize that the constituent studies do not all carry equal evidentiary force, and the strength of the ferroptosis claim should be read against the rigor of the experimental controls employed (Table 1). The most secure conclusions rest on a three-tier standard of proof: demonstration of the canonical biochemical signature (iron accumulation, GSH depletion and lipid peroxidation), pharmacological rescue by mechanistically independent ferroptosis inhibitors—ideally a radical-trapping antioxidant such as ferrostatin-1 together with an iron chelator such as deferiprone—and, most decisively, genetic loss- or gain-of-function manipulation of a pathway-defining node. The *S. aureus* [21] and *K. pneumoniae* [15,38] studies approach this standard, combining the biochemical signature with inhibitor rescue and, in the latter case, NCOA4 knockdown that both reverses ferroptosis and partially restores lactation. An intermediate tier comprises studies that show a consistent signature and inhibitor rescue but lack genetic confirmation, while the weakest claims derive from proteomic or metabolomic surveys that infer ferroptosis from upstream lipid- or iron-metabolic signatures alone [51,52]. Distinguishing these tiers is not merely pedantic: the omics-level associations are mechanistically suggestive and hypothesis-generating, but they cannot by themselves establish that ferroptosis is occurring, still less that it is causal, and they should be cited as motivating rather than confirmatory evidence until execution-stage markers are measured directly.

## 16. A Double-Edged Sword: Compartment-Specific Roles of Ferroptosis

**Figure 4:**
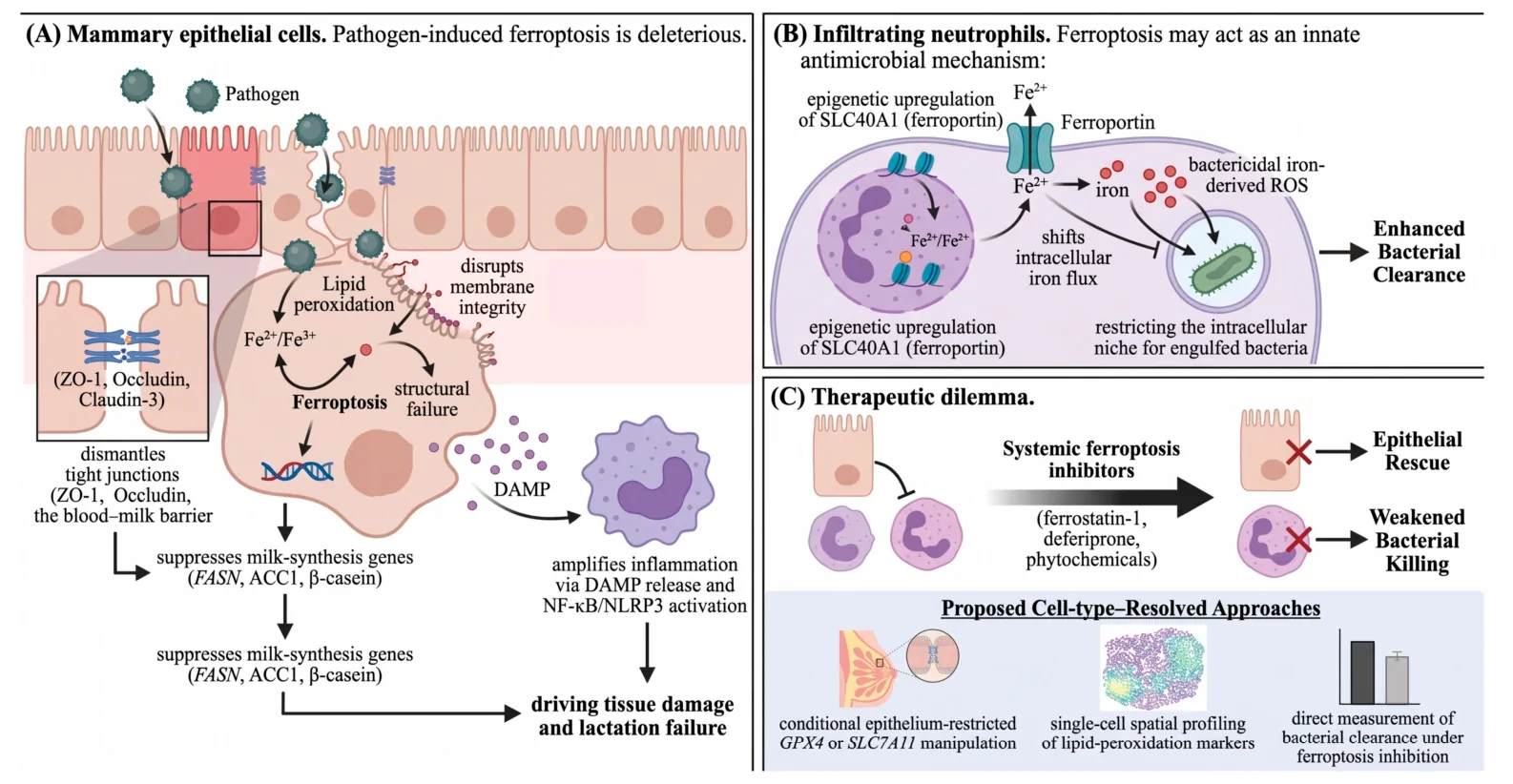
The double-edged sword: compartment-specific roles of ferroptosis in the infected mammary gland. The figure contrasts the pathological and potentially host-protective consequences of ferroptosis depending on the cellular compartment in which it is executed. (**A**) In mammary epithelial cells (left panel), pathogen-induced ferroptosis is unambiguously deleterious: iron-catalyzed lipid peroxidation disrupts membrane integrity, dismantles tight-junction complexes (ZO-1, Occludin, Claudin-3), breaches the blood–milk barrier, suppresses milk-synthesis genes (FASN, ACC1, β-casein), and amplifies inflammatory signaling through DAMP release and NF-κB/NLRP3 activation, collectively driving the tissue damage and lactation failure characteristic of clinical mastitis. (**B**) In infiltrating neutrophils (right panel), ferroptosis may serve as an innate antimicrobial mechanism: epigenetic upregulation of SLC40A1 (ferroportin) alters intracellular iron flux, and neutrophil ferroptosis could restrict intracellular bacterial survival by depriving engulfed pathogens of a viable intracellular niche, while also releasing bactericidal iron-derived ROS. (**C**) The therapeutic dilemma (center): systemically delivered ferroptosis inhibitors (ferrostatin-1, deferiprone, phytochemicals) act indiscriminately across both compartments, such that epithelial rescue may be achieved at the cost of blunted neutrophil-intrinsic killing; resolving this trade-off requires cell-type-resolved approaches, including conditional epithelium-restricted GPX4 or SLC7A11 manipulation, single-cell spatial profiling of lipid-peroxidation markers, and explicit measurement of bacterial clearance under ferroptosis inhibition. Created in BioRender (https://BioRender.com/6w538fw (accessed on 13 September 2026)).

Perhaps the most consequential unresolved question in this field is not whether ferroptosis occurs in mastitis, but where, in which cell type, and to whose benefit. The literature surveyed above treats ferroptosis almost exclusively as a pathogenic event, and within the mammary epithelium this framing is well justified: epithelial ferroptosis dismantles the blood–milk barrier, suppresses lactation and amplifies inflammation, and its inhibition is uniformly protective. Yet ferroptosis is fundamentally an ancient, cell-autonomous defense as much as a mode of injury, and at least one strand of the mastitis literature points to a markedly different role within the innate immune compartment. DNA hypomethylation in *E. coli*-infected mastitis neutrophils upregulate the iron exporter SLC40A1, and although the resulting iron flux could in principle sensitize these cells to ferroptosis, neutrophil ferroptosis may itself constrain intracellular bacterial survival—so that the same iron-dependent death program that destroys epithelium might, in the neutrophil, contribute to pathogen clearance [77]. Ferroptosis is, in this sense, a double-edged sword whose net effect on the host depends entirely on the cellular address at which it is executed (Figure 4).

This compartmental duality has a direct and underappreciated therapeutic implication. Every systemically delivered ferroptosis inhibitor evaluated to date—ferrostatin-1, deferiprone, and the great majority of the phytochemical and nutritional agents in Table 2—acts indiscriminately across cell types, and an intervention that rescues epithelial cells may simultaneously blunt a host-protective ferroptotic response in infiltrating neutrophils, potentially trading reduced tissue damage for impaired bacterial killing. The fact that several anti-ferroptotic agents nonetheless reduce bacterial load in vivo (for example, NCOA4 knockdown and GBMSC-ACE2 therapy both lowered viable counts [38,39]) suggests that, on balance, limiting epithelial destruction and preserving barrier integrity may outweigh any cost to neutrophil-intrinsic killing—but this balance has never been formally dissected. Resolving it will require cell-type-resolved approaches: conditional, epithelium-restricted manipulation of GPX4 or SLC7A11; single-cell or spatial profiling of lipid-peroxidation markers across the infected gland; and explicit measurement of bacterial clearance under ferroptosis inhibition. Until such studies are performed, the prevailing assumption that ferroptosis inhibition is unambiguously beneficial in mastitis should be regarded as a well-supported working hypothesis for the epithelium rather than an established principle for the gland as a whole. Framing the problem this way—as the search for the cell-type-specific and dose-dependent window in which ferroptosis modulation maximizes barrier protection without compromising antimicrobial defense—may prove more productive than the continued accumulation of single-agent protection studies.

## 17. Limitations and Knowledge Gaps

A recurring limitation across this literature is that several of the implicated mechanisms—particularly those drawn from proteomic and metabolomic surveys—infer ferroptosis from upstream metabolic signatures without direct measurement of GPX4 activity, lipid peroxidation or labile iron [51,52,60]. Direct interrogation of these execution-stage markers, ideally combining quantification of oxidized phospholipid species, the labile iron pool and GPX4 enzymatic activity with genetic loss- and gain-of-function controls, represents an important next step in establishing ferroptosis as a definitive and targetable driver of mastitis pathogenesis. Beyond this measurement gap and the compartment-specific considerations discussed in Section 12, much of the current evidence derives from murine models, immortalized cell lines (MAC-T), or short-term primary cultures, and the extent to which these recapitulate the chronic, polymicrobial, and host-genetically variable reality of field mastitis remains incompletely defined. The predominance of single-pathogen, single-timepoint challenge models also leaves the temporal dynamics of ferroptosis across the course of natural infection—and its interaction with co-infecting organisms—largely unexplored.

## 18. Future Perspectives

The convergence of multiple independent laboratories on the system X_c_^−^/GSH/GPX4 axis and on NCOA4-mediated ferritinophagy provides a firm foundation for the rational design of anti-ferroptotic therapeutics for mastitis. Several priorities emerge. First, the ferroptosis-associated genes repeatedly identified across transcriptomic studies—notably *HMOX1*, *SAT1*, *SLC40A1* and *FTH1*—warrant validation as candidate markers in dairy cattle breeding programs aimed at enhancing mastitis resilience [23,29,77]. Second, the natural bioactive compounds and nutritional factors cataloged here, many of which combine antimicrobial, anti-inflammatory and anti-ferroptotic activities, represent attractive antibiotic-sparing candidates whose pharmacokinetics, milk-residue profiles and efficacy in naturally infected herds now require rigorous evaluation. Third, advanced modalities—selenium nanoparticles, milk-derived extracellular vesicles, and engineered ACE2-overexpressing mesenchymal stem cells—offer multi-target precision but require assessment of scalability, safety, and cost-effectiveness at the production scale [39,53,64]. Finally, integrating ferroptosis biology with the gut– and rumen–mammary axes opens the prospect of dietary and microbiome-based prophylaxis that pre-empts the LPS-driven ferritinophagy–ferroptosis cascade before clinical disease develops [43,49].

## 19. Conclusions

Ferroptosis has emerged as a prominent and mechanistically coherent candidate driver of mammary epithelial injury in mastitis. Across phylogenetically diverse pathogens, including *S. aureus*, *E. coli*, *K. pneumoniae*, *S. uberis* and Group B *Streptococcus*, as well as sterile inflammatory and environmental insults, the available evidence indicates that the disease frequently engages a shared three-pillar program comprising iron liberation, lipid-substrate enrichment and antioxidant-axis collapse. This program is executed through ferritinophagy, mitophagy and chaperone-mediated autophagy, and is regulated by the Nrf2-controlled SLC7A11/GSH/GPX4 system. Consistent protection observed with mechanistically diverse agents that converge on this axis, together with the reproducible efficacy of the reference inhibitors ferrostatin-1 and deferiprone, supports ferroptosis as a biologically relevant therapeutic target in this disease. Realizing this promise will require direct measurement of execution-stage ferroptosis markers, careful attention to cell-type-specific and host-protective roles, and validation in naturally infected dairy herds. With these caveats addressed, the targeting of ferroptosis—whether through phytochemicals, nutritional supplementation, selenium nanomedicine, biologics or marker-assisted breeding—offers a compelling, antimicrobial-sparing frontier for the prevention and treatment of one of the dairy industry’s most intractable diseases.

## Figures and Tables

**Table 1:** Mastitis-associated triggers and the ferroptotic mechanisms they engage in mammary epithelial cells.

Trigger/Stimulus	Model System	Ferroptotic Mechanism Engaged	Key Molecular Readouts	Direct Ferroptosis Evidence	References
*Staphylococcus aureus*	Murine mastitis; primary MMECs	ER stress (PERK–eIF2α–ATF4–CHOP) → autophagic ferritin degradation; ROS feed-forward loop	↑ MDA, ↑ Fe^2+^; ↓ GSH, ↓ GPX4, ↓ ferritin; Fer-1/DFO protective	Direct	[21]
*Escherichia coli*	Bovine MECs (bMECs)	Wnt/β-catenin-driven mitophagy; GPX4 suppression	Rapamycin ↑, chloroquine ↓, foscenvivint ↓ ferroptosis	Direct	[37]
*Klebsiella pneumoniae*	bMECs; murine tissue	NCOA4-mediated ferritinophagy; Nrf2/xCT/GPX4 suppression	↑ Fe^2+^, ↑ 4-HNE, ↑ ROS; ↓ GPX4, ↓ SLC7A11; NCOA4 KD rescues	Direct	[15,38]
*Streptococcus uberis*	Goat MECs (GMECs); in vivo	AngII/AT1R/NOX2 ROS → chaperone-mediated autophagy degrades GPX4	↑ ACSL4, ↑ COX2; ↓ GPX4; ACE2/Fer-1 protective	Direct	[39,40]
Group B *Streptococcus* (GBS)	Murine mastitis	TfR1/NCOA4 iron loading; SLC7A11/GPX4 depletion	↑ iron, ↑ PTGS2, ↑ ACSL4; quercetin reverses	Direct	[41]
*Pseudomonas aeruginosa* (pyocyanin)	bMECs	Nrf2/HO-1 suppression; p53 activation represses SLC7A11	↑ MDA, ↑ ROS; ↓ GSH-Px, ↓ T-AOC; worsened by LPS	Partial	[42]
LPS/endotoxin	MAC-T cells; murine tissue	TLR4/CD36/FAK/mTORC1 → IL-6 → ferroptosis; Nrf2/GPX4 collapse	↑ Fe^2+^, ↑ ROS, ↑ MDA; RSL3-reversible; impaired milk synthesis	Direct	[22,43,44]
Interleukin-6 (IL-6)	Goat MECs	USP14-mediated IL-6 stabilization; NRF2 suppression	↑ Fe^2+^, ↑ ROS, ↑ MDA, ↓ GSH; Fer-1 reverses	Direct	[45]
Heat stress	bMECs	Iron/lipid-ROS rise; methionine restores Nrf2 axis	↑ LIP, ↑ MDA; ↓ GPX4, ↓ SLC7A11, ↓ FTH1	Direct	[46]
ZnO nanoparticles	Lactating mice	NF-κB/MAPK inflammation + iron/lipid oxidation	↑ iron, ↑ lipid oxidation; tight-junction loss	Partial	[47]
Glyphosate (herbicide)	MAC-T cells	Ferroptosis upstream of inflammation and barrier loss	↑ Fe^2+^, ↑ ROS, ↓ GSH; Fer-1 protective	Direct	[48]
*STING* activation	MMECs (*S. aureus*)	STING–FASN–ACSL4 de novo lipogenesis fuels peroxidation	↑ lipid peroxides; GSH depletion; STING inhibition reverses	Direct	[25]
Ruminal dysbiosis (high-concentrate diet)	Dairy goats → mouse transfer	LPS ↑ TFR1, ferritinophagy, STEAP3 Fe^3+^→Fe^2+^; Xc^−^/GSH/GPX4 loss	Fenton ROS; lipid peroxides; Fer-1 alleviates	Direct	[49]
Bovine leukemia virus (BLV)	bMECs (metabolomics)	Unsaturated FA biosynthesis + vitamin B6 disruption; ↓ CoQ10	↑ EPA/DPA/DHA/AA-PUFAs; System Xc^−^ perturbation	Inferred	[50]
Clinical mastitis (proteome/lipidome)	Bovine mammary tissue	ACOT7/arachidonic acid metabolism expands PUFA substrate	↑ ACOT7, ↑ AA metabolites; execution markers not measured	Inferred	[51,52]

## Data Availability

No new data were created or analyzed in this study. Data sharing is not applicable.
